# Microbial d-xylonate production

**DOI:** 10.1007/s00253-012-4288-5

**Published:** 2012-08-09

**Authors:** Mervi H. Toivari, Yvonne Nygård, Merja Penttilä, Laura Ruohonen, Marilyn G. Wiebe

**Affiliations:** VTT, Technical Research Centre of Finland, P.O. Box 1000, 02044 VTT Espoo, Finland

**Keywords:** d-Xylonate, d-Xylose, Oxidation, d-Xylose dehydrogenase, Lignocellulosic hydrolyzate

## Abstract

d-Xylonic acid is a versatile platform chemical with reported applications as complexing agent or chelator, in dispersal of concrete, and as a precursor for compounds such as co-polyamides, polyesters, hydrogels and 1,2,4-butanetriol. With increasing glucose prices, d-xylonic acid may provide a cheap, non-food derived alternative for gluconic acid, which is widely used (about 80 kton/year) in pharmaceuticals, food products, solvents, adhesives, dyes, paints and polishes. Large-scale production has not been developed, reflecting the current limited market for d-xylonate. d-Xylonic acid occurs naturally, being formed in the first step of oxidative metabolism of d-xylose by some archaea and bacteria via the action of d-xylose or d-glucose dehydrogenases. High extracellular concentrations of d-xylonate have been reported for various bacteria, in particular *Gluconobacter oxydans* and *Pseudomonas putida*. High yields of d-xylonate from d-xylose make *G. oxydans* an attractive choice for biotechnical production. *G. oxydans* is able to produce d-xylonate directly from plant biomass hydrolysates, but rates and yields are reduced because of sensitivity to hydrolysate inhibitors. Recently, d-xylonate has been produced by the genetically modified bacterium *Escherichia coli* and yeast *Saccharomyces cerevisiae* and *Kluyveromyces lactis*. Expression of NAD^+^-dependent d-xylose dehydrogenase of *Caulobacter crescentus* in either *E. coli* or in a robust, hydrolysate-tolerant, industrial *Saccharomyces cerevisiae* strain has resulted in d-xylonate titres, which are comparable to those seen with *G. oxydans*, at a volumetric rate approximately 30 % of that observed with *G. oxydans*. With further development, genetically modified microbes may soon provide an alternative for production of d-xylonate at industrial scale.

## Introduction

**Fig. 1:**
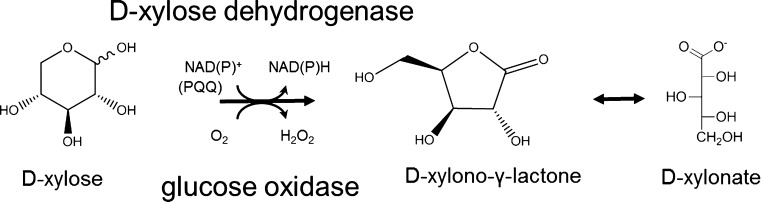
Formation of d-xylonate from d-xylose by NAD(P)^+^ or PQQ-dependent xylose dehydrogenases or glucose oxidase

Sugar acids are currently generating considerable interest because of their potential as platform chemicals and particularly their use as precursors in the manufacture of biomass derived plastics. d-Xylonic acid (Fig. 1), derived from the hemicellulose sugar d-xylose, has applications similar to d-gluconic acid and could serve as a d-gluconic acid substitute, but would be produced from non-food carbohydrate. d-Xylonic acid has been used in dispersal of concrete (Chun et al. 2006), in the production of copolyamides (Zamora et al. 2000) and as a precursor for 1,2,4-butanetriol synthesis (Niu et al. 2003). Several other applications for d-xylonic acid have been patented.

**Fig. 2:**
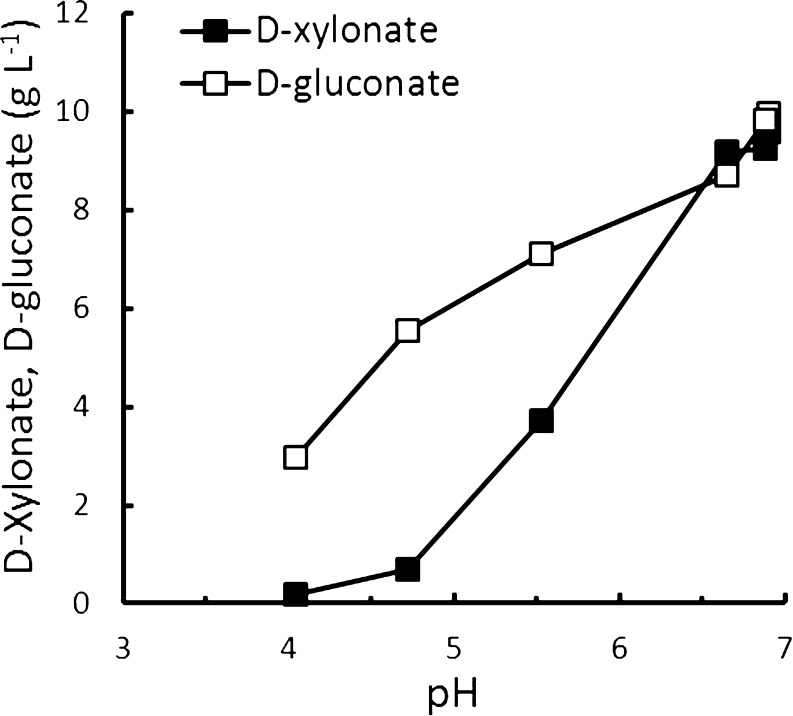
Production of d-xylonate and d-gluconate by *Aspergillus niger* ATCC1015 after 79 h in defined medium with 45 g d-xylose l^−1^ and 10 g d-glucose l^−1^ as carbon source. Medium was buffered with 0.1 to 2.0 % (*w*/*v*) CaCO_3_, and average pH over 79 h is shown

Microbial production of d-xylonate was recognised already at the end of the nineteent century (Bertrand 1898, cited in Lockwood and Nelson 1946) and many species of *Pseudomonas*, *Acetobacter*, *Aerobacter*, *Gluconobacter*, *Erwinia* and related genera have been shown to produce d-xylonate (reviewed in Buchert 1990). Periplasmic d-xylose and d-glucose dehydrogenases use the pyrroloquinoline quinol (PQQ) prosthetic group to transfer electrons to cytochrome c in the respiratory chain, with a corresponding accumulation of d-xylonolactone or d-xylonate in the medium (Galar and Boiardi 1995; Hardy et al. 1993). d-Xylonolactone is the immediate product of the dehydrogenases, but the lactone generally opens spontaneously or with the aid of lactonase produced by the same species (Buchert and Viikari 1988). Some bacteria and also archaea metabolise d-xylonic acid further via non-phosphorylative d-xylose metabolic pathways (Weimberg 1961; Dahms 1974). Cytoplasmic NAD(P)^+^-dependent d-xylose dehydrogenases oxidise d-xylose to d-xylonolactone (Johnsen and Schönheit 2004; Johnsen et al. 2009; Stephens et al. 2007), which is cleaved by lactonase to d-xylonate in the cytoplasm. d-Xylonate may be dehydrated to produce 2-keto-3-deoxy pentanoate, which is further dehydrated and reduced to α-ketoglutarate or cleaved by an aldolase to pyruvate and glycolaldehyde. There are also some reports of yeast and other fungi producing d-xylonic acid (Suzuki and Onishi 1973; Kiesling et al. 1962; Kanauchi and Bamforth 2003), although only one gene coding for d-xylose dehydrogenase has been identified in fungal species (Berghäll et al. 2007). Production of d-xylonate from d-xylose by d-glucose oxidase has also been described (Pezzotti and Therisod 2006; Chun et al. 2006) and *Aspergillus niger* produces d-xylonate when cultivated in suitable conditions (Fig. 2).

Recently, various yeast strains as well as the bacterium *Escherichia coli* have been engineered to produce d-xylonate, by the introduction of genes encoding d-xylose dehydrogenase (Toivari et al. 2010; Nygård et al. 2011; Liu et al. 2011). Gene sequences for several putative d-xylonolactonases have recently been identified (Johnsen et al. 2009; Stephens et al. 2007; Brouns et al. 2006), but the enzymes have not been studied. The mechanism of transport of either the linear or the lactone form of d-xylonate from strains with intracellular d-xylonate production is unknown.

In addition to the microbial production described in this review, d-xylonate can be produced via enzymatic (Pezzotti and Therisod 2006), electrochemical (Jokic et al. 1991) or chemical oxidation (Isbell and Hudson 1932). d-Xylonic acid can also be found in acid sulphite pulping liquor of hardwood (Samuelson and Simonson 1962; Pfister and Sjöström 1977). However, an efficient separation method to obtain d-xylonate from pulping liquor has not been established.

Although a variety of applications for d-xylonic acid have been patented, one of which includes a method for production of crude d-xylonic acid from plant biomass hydrolysate (Chun et al. 2003), bulk production of d-xylonic acid is limited. This review describes the current state in microbial production of d-xylonate with bacteria and fungi.

## Bacterial d-xylonate production

### Yields and conversion rates

Of the numerous bacteria described as producers of d-xylonate, species of *Pseudomonas* (Lockwood and Nelson 1946; Buchert et al. 1986), *Gluconobacter* (Buchert 1990), *Micrococcus* (Ohsugi et al. 1970) and *Enterobacter* (Ishizaki et al. 1973) have been the most productive (Table 1). High yields of d-xylonate are generally associated with poor or no conversion of d-xylose to biomass.

**Table 1:** d-Xylonate production with *G. oxydans*, *Pseudomonas* species, and *Enterobacter cloacea*, *A. niger*, and engineered strains of *Escherichia coli*, *S. cerevisiae* and *K. lactis*

Species	d-Xylose (g l^−1^)	d-Xylonate (g l^−1^)	Yield_P/S_ (g g^−1^)	Volumetric productivity (g l^−1^ h^−1^)	Specific productivity [g (g biomass)^−1^ h^−1^]	pH	Biomass (g l^−1^)	Process	References
*G. oxydans* (ATCC621)	100	109	1.1	2.5	~1.5	5.5	1.7	Batch	Buchert (1990)
*G. oxydans* (ATCC621)	100	107	1.1	2.2	~1.5	4.5	1.3	Batch	Buchert (1990)
*G. oxydans* (ATCC621)	46	51	1.1	1.8	6	5.5	0.2	Batch	VTT
*G. oxydans* (ATCC621)	40	41	1.0	1.0	4	3.5	0.2	Batch	VTT
*G. oxydans* (ATCC621)	40	37	1.0	1.5	2.8	5.5	0.5	Continuous *D* = 0.04 h^−1^	VTT
*P. fragi* ATCC4973	150	162	1.1	1.4	0.2	6.5	6.9	Batch	Buchert and Viikari (1988)
*P. putida*	~0.4	~0.4	~1	~1.9	~0.7	6.8	2.9	Continuous *D* = 0.2 h^−1^	Hardy et al. (1993)
*E. cloacea*	200	190	~1	1.6		6.5	nd	Batch	Ishizaki et al. (1973)
*E. coli*	40	39	1.0	1.1	0.14	7.0	~8	Batch	Liu et al. (2011)
*S. cerevisiae Xyd1*	20	4	0.4	0.03	0.007	5.5	4.6	Batch	Toivari et al. (2010)
*S. cerevisiae* SUS2DD	23	3	0.4	0.02	0.006	5.5	5.3	Batch	Toivari et al. (2012)
*S. cerevisiae xylB*	23	17	0.8	0.23	0.06	5.5	5	Batch	Toivari et al. (2012)
*S. cerevisiae* B67002 *xylB*	49	43	0.8	0.44	0.06	5.5	7	Batch	Toivari et al. (2012)
*K. lactis Xyd1*	40	19	0.6	0.16	0.03	5.5	6	Batch	Nygård et al. (2011)
*K. lactis Xyd1* Δ*XYL1*	23	8	0.4	0.13	0.01	5.5	9	Batch	Nygård et al. (2011)
*A. niger* ATCC1015	45	10	0.8	0.12		>5.5	nd	Batch	VTT

For production potential of other bacteria, see (Buchert 1990).

*nd* = no data, *VTT* unpublished data from VTT, M.G. Wiebe personal communication

**Fig. 3:**
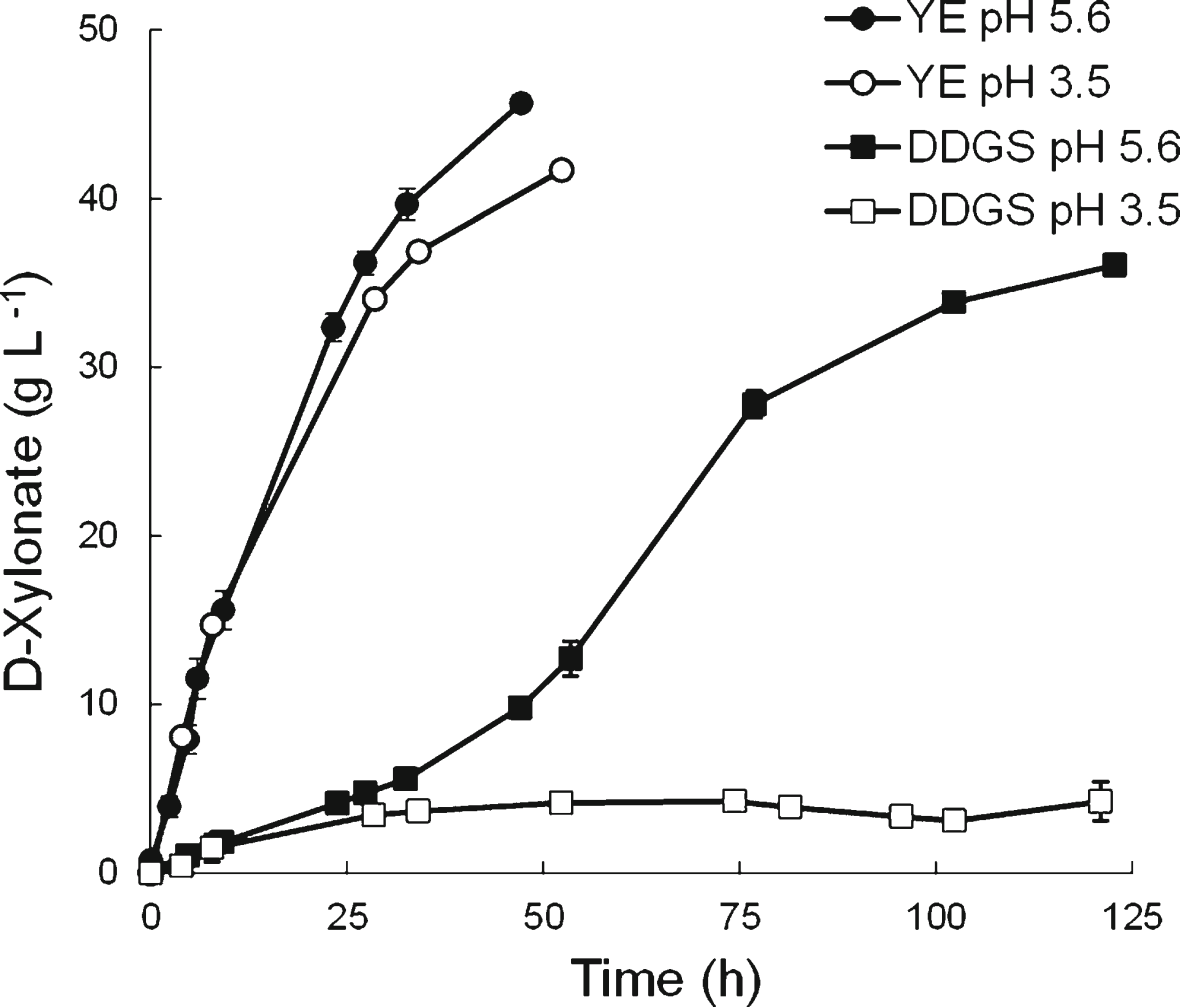
d-Xylonate production by *Gluconobacter oxydans* ATCC621 from d-xylose in YE supplemented defined medium with 45 g d-xylose l^−1^ at pH 5.6 (*filled circle*) or pH 3.5 (*empty circle*) and from acid hydrolysed DDGS at pH 5.6 (*filled square*) or pH 3.5 (*empty square*). *Error bars* represent ±SEM for duplicate cultures

Although the pH optimum of the *Gluconobacter oxydans* d-xylose dehydrogenase is 6, d-xylonate has been produced at pH 4.5 (Buchert 1990) and even at pH 3.5 (Fig. 3, Table 1). Production rates are approximately 2 g d-xylonate l^−1^ h^−1^ at pH 4.5–6.5, even when biomass concentration is low (0.2 g biomass l^−1^) and no cell growth occurs (Table 1, Buchert 1990). We have observed specific production rates up to 12 g d-xylonate (g biomass)^−1^ h^−1^ at pH 5.6.

Since *G. oxydans* requires complex growth medium and efficiently converts most sugars to acids rather than biomass, other species may be more cost effective for d-xylonate production. *Pseudomonas fragi* ATCC4973 produces d-xylonate at similar volumetric rates to *G. oxydans*, but at lower specific rate (Table 1, Buchert and Viikari 1988), and production is more sensitive to pH and hydrolysate inhibitors (Buchert et al. 1986, 1988). Various other bacteria also produce d-xylonate and some such as *Gluconoacetobacter diazotrophicus*, which has been considered as an alternative to *Gluconobacter* for d-gluconate production because of its minimal nutritional requirements and low pH tolerance (Attwood et al. 1991) could also be considered for d-xylonate production.

The first example of bacteria engineered for d-xylonate production was recently described by Liu et al. (2011). By introducing a d-xylose dehydrogenase encoding gene, *xylB* from *Caulobacter crescentus*, into *E. coli* strain W3110 and by blocking the endogenous pathways for d-xylose and d-xylonate metabolism, they were able to produce 39 g l^−1^ d-xylonic acid from 40 g l^−1^ d-xylose in a batch process (Table 1).

**Fig. 4:**
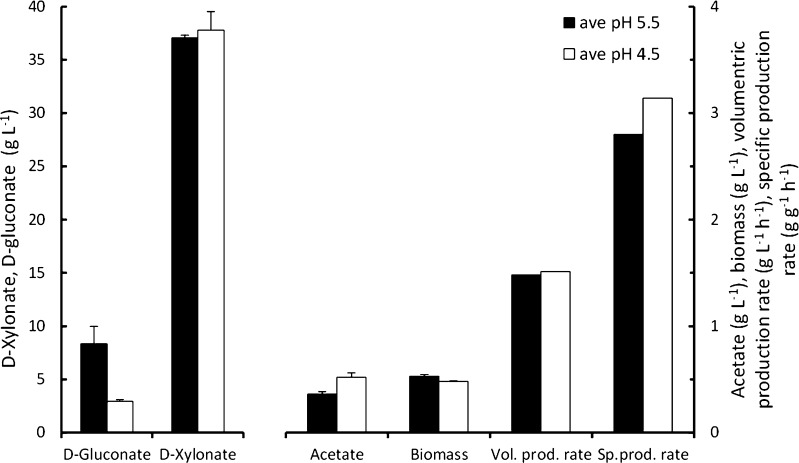
d-Gluconate, d-xylonate, acetate and biomass production, volumetric d-xylonate production rate and specific d-xylonate production rate by *Gluconobacter oxydans* ATCC621 in chemostat culture with YE supplemented defined medium containing 10 g d-glucose l^−1^ and 40 g d-xylose l^−1^ at *D* = 0.04 h^−1^, pH 5.5 or 4.5. *Error bars* represent ±SEM for triplicate (pH 5.5) or duplicate (pH 4.5) samples

Although continuous production of d-xylonate has not been reported, continuous production of d-gluconic (e.g. Attwood et al. 1991), 2-keto-l-gulonic (e.g. Takagi et al. 2009) and 2,5-diketogluconic (e.g. Buse et al. 1992a, b) acids have been described. Hardy et al. (1993) described the production of d-xylonate at a rate of ~4 mmol (g biomass)^−1^ h^−1^ as a by-product for enhanced biomass yield of *Pseudomonas putida* on d-glucose or lactate at *D* = 0.2 h^−1^, pH 6.8 (Table 1). With *G. oxydans*, we have observed continuous production of d-xylonate with *G. oxydans* at a rate of 1.5 g d-xylonate l^−1^ h^−1^ at *D* = 0.04 h^−1^ with 40 g d-xylose l^−1^ and 20 g d-glucose l^−1^ at pH 5.5 (Fig. 4, Table 1). In addition, d-gluconate, acetate and biomass were produced. Continuous production at pH 4.5 was also possible (Fig. 4). Conditions for d-xylonate production in fed-batch cultures have not been reported.

### Hydrolysate

Lignocellulosic waste biomass would provide an economic raw material for d-xylonate production, and several studies have been carried out on the conversion of d-xylose to d-xylonate in hemicellulose hydrolysates (Chun et al. 2006; Buchert et al. 1988). These are summarised in Table 2. *G. oxydans* was found to be more tolerant to toxins in biomass hydrolysate than *P. fragi*, but growth and d-xylonate production were still inhibited by high concentrations of lignocellulosic hydrolysate. Pre-treatment by diethylether extraction, adsorption on mixed bed resin or ion exclusion chromatography enabled conversion of d-xylose in hydrolysate to d-xylonate by *G. oxydans*, with the biggest improvements seen when treated by ion exclusion chromatography (Table 2).

**Table 2:** Production of d-xylonate from lignocellulosic hydrolysates with *G. oxydans* ATCC621

d-Xylose (g l^−1^)	d-Xylonate (g l^−1^)	Volumetric productivity (g d-xylonate l^−1^ h^−1^)	Hydrolysate	References
25	~13	0.2	Birchwood, steam	Buchert et al. (1988)
25	~22	0.3	Birchwood, steam, ether extracted	Buchert et al. (1988)
100	~88	1.2	Birchwood, steam, ion exclusion	Buchert et al. (1990)
~45	~48	~0.5	Birch spent sulphite liquor	Chun et al. (2006)
39	17	0.4	Wheat straw (ABNT), C5 fraction derived^a^ from steam pre-treatment	Turkia et al. (2010)
na	54	1.1	Wheat straw (ABNT), C5 fraction derived^a^ from steam pre-treatment, overlimed	VTT
35	35	0.6	DDGS (ABNT), acid hydrolysed	VTT
25	13	0.3	DDGS (ABNT), acid hydrolysed, continuous at *D* = 0.03 h^−1^	VTT
23	5	0.1	DDGS (ABNT), acid hydrolysed, overlimed, continuous at *D* = 0.02 h^−1^	VTT

When described, cultures were maintained at pH 5.5–6.5

*ABNT* Abengoa Bioenergia Nuevas Tecnologias, *VTT* unpublished data from VTT, M.G. Wiebe personal communication, *na* not available

^a^Provided by Dr. Robert Bakker, Wageningen University & Research Centre

**Fig. 5:**
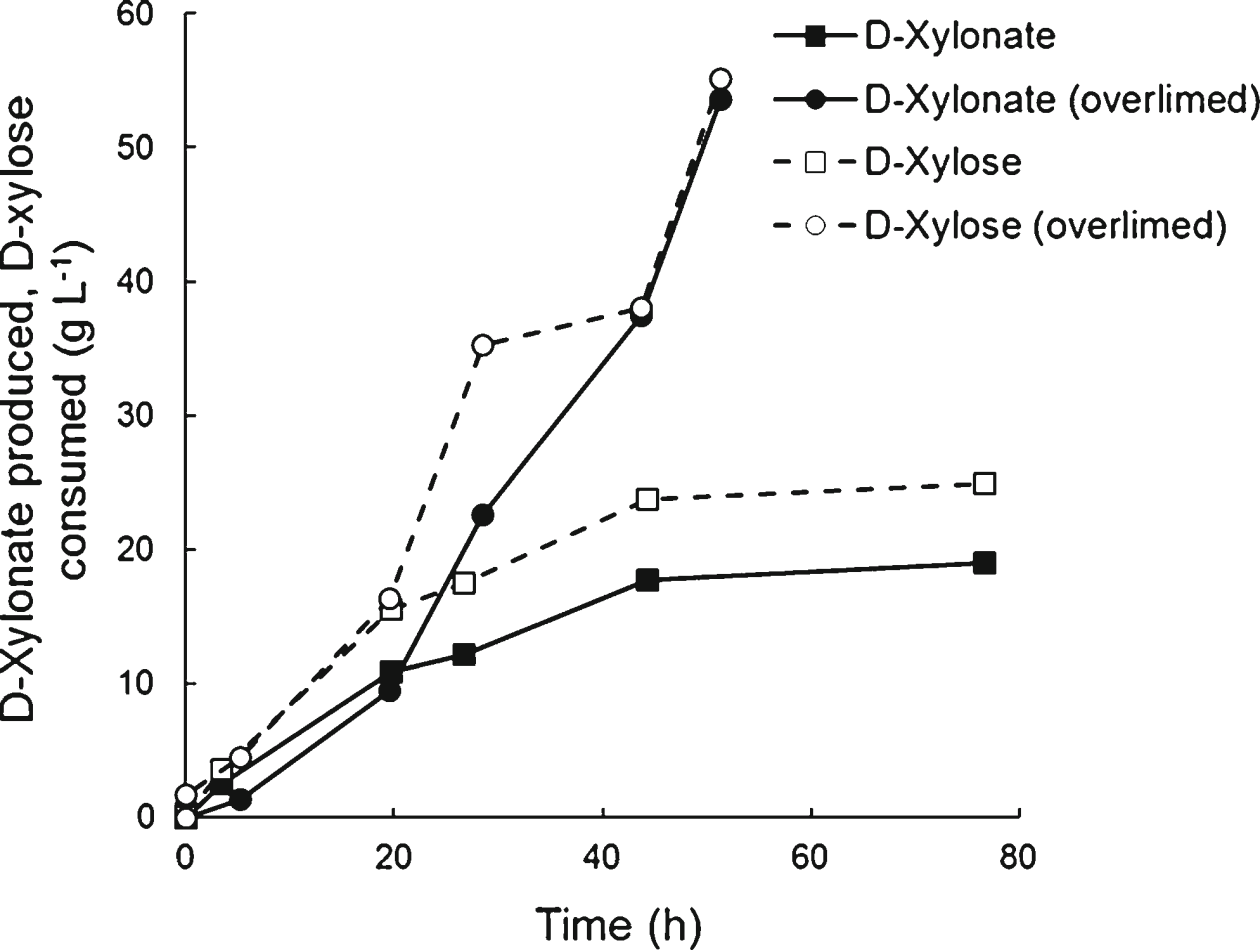
d-Xylonate produced (solid symbols) and d-xylose consumed (*open symbols*) by *Gluconobacter oxydans* ATCC621 in pre-treated wheat straw derived hydrolysate (C5 fraction), with (*circles*) or without (*squares*) overliming, and supplemented with 5 g yeast extract l^−1^ at pH 5.6, 30 °C. The hydrolysate contained d-xylose, d-glucose, L-arabinose, and acetate. d-Xylonate measurements in untreated wheat straw hydrolysate are shown in Turkia et al. (2010)

Turkia et al. (2010) also observed a low rate for the conversion of d-xylose to d-xylonate by *G. oxydans* ATCC621 (E97003) in a pentose-rich hydrolysate derived from wheat straw, with incomplete conversion of the d-xylose and a low yield of ~0.7 g d-xylonate (g d-xylose consumed)^−1^. Overliming (Mohagheghi et al. 2006) the hydrolysate to remove some of the aromatic and aliphatic compounds was sufficient to enable full conversion of the hydrolysate [yield 1.0 g d-xylonate (g d-xylose consumed)^−1^] and to improve the production rate to 1.1 g d-xylonate l^−1^ h^−1^ with an inoculum of only 1.0–1.5 g biomass l^−1^ (Fig. 5). The rate was thus only slightly lower than that observed with pure d-xylose and similar to that obtained by hydrolysate treated with ion exclusion chromatography. In contrast, overliming acid hydrolysed dried distillers grain solids (DDGS) from Abengoa Bioenergia Nuevas Tecnologias (ABNT, Spain) resulted in poorer conversion of the d-xylose to d-xylonate than in the untreated hydrolysate (Table 2).

Chun et al. (2006) found that *G. oxydans* was able to completely convert d-xylose in diluted spent sulphite liquor even though no cell growth occurred. Conversion rates with high cell density (~4.6 g biomass l^−1^) were comparable to those in ether-extracted birchwood hydrolysate and untreated wheat straw hydrolysate (Table 2, Chun et al. 2006).

In continuous flow culture at *D* = 0.03 h^−1^, d-xylonate was produced by *G. oxydans* at a rate of 0.32 g d-xylonate l^−1^ h^−1^ for up to 3 days (Table 1). However, only ~50 % of the d-xylose in the hydrolysate was converted to d-xylonate, and the cells were being washed out. Approximately 76 % d-glucose in the hydrolysate was converted to d-gluconate.

## Recombinant yeast for d-xylonate production

### *S. cerevisiae* and the choice of d-xylose dehydrogenase

Toivari et al. (2010) described the production of d-xylonate by *S. cerevisiae* expressing an NADP^+^-dependent xylose dehydrogenase from *T. reesei*. The engineered *S. cerevisiae* strains produced up to 3.8 g d-xylonate l^−1^ (Table 1). Xylitol (4.8 g l^−1^) was the primary by-product and could be significantly reduced by deleting the aldose reductase encoded by *GRE3* (Toivari et al. 2010). Although this demonstrated the feasibility of producing d-xylonate with yeast, the titres and rates were low compared to those obtained with bacteria (see above) or for other acids produced in *S. cerevisiae*, e.g. lactate >100 g l^−1^ (Sauer et al. 2010).

Initial attempts at redox engineering to improve NADP^+^-recycling did not improve d-xylonate production (Toivari et al. 2010), and the activity of alternative d-xylose dehydrogenases was assessed in *S. cerevisiae* (Toivari et al. 2012, Table 1). The *C. crescentus xylB* encoded NAD^+^-dependent d-xylose dehydrogenase was found to have high activity in *S. cerevisiae*, and strains expressing *xylB* produced more d-xylonate (17 ± 2 g l^−1^) at a higher rate than the X*yd1* expressing strain (Table 1, Toivari et al. 2012). In addition to high activity, the xylB had high specificity for d-xylose (Toivari et al. 2012). This increase in d-xylonate production with *xylB* compared to *Xyd1* presumably reflects the higher activity of the dehydrogenase in the cytoplasm. However, it may also indicate that production of excess NADH, which can be oxidised in the electron transport chain to generate energy, is preferable to production of excess NADPH. Another NADP^+^ requiring d-xylose dehydrogenase, SUS2DD from pig liver, which was successfully expressed in *S. cerevisiae*, showed similar activity and production characteristics to the *T. reesei Xyd1* (Table 1, Toivari et al. 2012).

Expression of *xylB* in the industrial *S. cerevisiae* strain B67002 enabled the production of higher concentrations of d-xylonate (e.g. 43 g l^−1^) than with the lab strain, at rates approaching 0.5 g l^−1^ h^−1^, i.e. 25–30 % those observed with *Gluconobacter* and *Pseudomonas* spp. (Table 1; Toivari et al. 2012).

Only limited research has been carried out on the environmental conditions that are required for good d-xylonate production by *S. cerevisiae*. Production has primarily been characterised at pH 5.5. d-Xylonate could also be produced at pH 3, but production and cell viability were reduced compared to that observed at pH 5.5 (Toivari et al. 2012). Similarly, low productivity at low pH has also been observed with lactic acid production (Porro et al. 1999). d-Xylonate accumulated intracellularly (Toivari et al. 2012), revealing a potential need to engineer d-xylonate transport. d-Xylonate production has been shown to be an energy requiring process, with production essentially stopping once metabolisable carbon has been consumed, but resuming when additional co-substrate is added (Toivari et al. 2010). Since most biomass hydrolysates, even C5-enriched fractions, contain some C6 sugars, co-substrate would be at least partly provided from the biomass hydrolysate. Because d-xylonate production produces NADH (or NADPH), which needs to be oxidized, ultimately by channelling electrons to oxygen, the process must be aerobic. However, the energy produced in the reduction of oxygen will provide energy for the process, including d-xylonate transport (if active), pH homeostasis and cell maintenance.

When a putative d-xylono-lactone lactonase *xylC* from *C. crescentus* was expressed together with *xylB* in *S. cerevisiae*, increased lactonase activity was observed by NMR. More extracellular d-xylonate was initially produced than with cells lacking *xylC* at both pH 5.5 and 3 (Toivari et al. 2012). The lactonase-expressing strain also sustained higher production at pH 3. However, expression of the lactonase encoding gene decreased cell vitality and viability when d-xylonate was produced at pH 3.0 (Toivari et al. 2012).

### Alternative yeast for d-xylonate production

d-Xylonate production has also been demonstrated with the d-xylose-utilising yeast *K. lactis*. d-Xylonate was produced in *K. lactis* with the Xyd1 enzyme from *T. reesei*, which is NADP^+^-dependent and has relatively low activity. Although the activity levels were similar to those observed in *S. cerevisiae*, *K. lactis* produced more d-xylonate (6.3 ± 0.1 g l^−1^) at a higher rate (Nygård et al. 2011), compared to *S. cerevisiae* expressing the same gene. Increasing the substrate concentration led to higher productivity (19 ± 2 g l^−1^ at rates of 0.16 ± 0.01 g l^−1^ h^−1^, Table 1; Nygård et al. 2011), whereas the equivalent *S. cerevisiae* strains did not increase d-xylonate production when provided higher d-xylose concentrations. The natural ability to utilize d-xylose not only may benefit d-xylonate production by decreasing the need for added co-substrate but also decreases the overall yield and thus should be optimized to support good productivity without substantial loss in yield.

In *K. lactis* the deletion of the xylose reductase (encoded by *XYL1*) resulted not only in less xylitol production compared to the reductase containing strain but also increased d-xylonate production (Nygård et al. 2011), in contrast to the effect of deleting *GRE3* from *S. cerevisiae*, which only reduced xylitol production (Toivari et al. 2010). With *K. lactis*, oxygen provision affected the conversion of d-xylose to d-xylonate, xylitol or biomass. Metabolism of d-xylose was most efficient with high oxygen provision (12 mmol O_2_ l^−1^ h^−1^), but even with low oxygen concentration (6 mmol O_2_ l^−1^ h^−1^), no loss in d-xylonate titre or production rate occurred.

*K. lactis* is not particularly tolerant to biomass hydrolysates, but its good d-xylonate production ability demonstrates the potential benefit of producing d-xylonate in a d-xylose-utilising non-*Saccharomyces* yeast.

## The prospects for future microbial d-xylonate production

Although the non-engineered bacteria are very efficient at producing d-xylonate, neither commercial production has been described, nor have cost effective, large-scale separation processes been developed. The current methods, e.g., precipitation or ion exchange, are for small-scale preparations from relatively pure solutions (Liu et al. 2011; Buchert et al. 1986; Devos and Huchette 1981) and would not be adequate for bulk production of d-xylonic acid required for use as a platform chemical, e.g., for polymer or hydrogel production. Since there is no historic market for d-xylonate, there has been no driving force to develop large-scale production. However, with the increasing need to replace petrochemicals and compounds derived from d-glucose, such as d-gluconate, with alternative chemicals, interest in large-scale production and purification of d-xylonate will grow.

Production of d-xylonate by *G. oxydans* has been limited by complex nutritional requirements and low biomass production, requiring costly inoculum development, even if d-xylonate could then be produced from biomass hydrolysates. Several patents improving biomass production by *G. oxydans* have been published (Zhao et al. 2011; Yuan et al. 2009; Shingoh 2009), but strategies that disrupt the peri- or cytoplasmic glucose dehydrogenases (Shingoh 2009) are expected to also reduce the d-xylonate production rate. *P. fragi* would provide more robust inoculum development, but would require more extensive treatment to remove inhibitors from the biomass hydrolysates than *G. oxydans* (Buchert et al. 1988). *G. diazotrophicus* may be a reasonable alternative (Attwood et al. 1991), but has not been evaluated in hydrolysate. Another major concern with these bacteria is the range of acidic products produced from the compounds present in lignocellulosic hydrolysates and their separation costs.

Genetically engineered bacteria and yeast now provide new alternatives to the non-engineered bacteria for large-scale production of d-xylonate and are likely to be developed further. The engineered *E. coli* strain provides benefits in having a fast specific growth rate, efficient generation of inoculum, and low nutrient requirements. Yeast such as *S. cerevisiae* and *K. lactis* also have good growth and low nutritional requirements. *S. cerevisiae* and several other yeast (e.g. Kwon et al. 2011) additionally offer good tolerance to the various inhibitors found in lignocellulosic hydrolysates, as well as tolerance to low pH conditions and even the capacity for acid production at low pH (cf. lactic acid production at pH 3, Suominen et al. 2009). The development of genetically engineered production strains opens new doors for the development of robust industrial processes for d-xylonic acid production.
